# Double-arc volumetric modulated therapy improves dose distribution compared to static gantry IMRT and 3D conformal radiotherapy for adjuvant therapy of gastric cancer

**DOI:** 10.1186/s13014-015-0420-x

**Published:** 2015-05-19

**Authors:** Tao Zhang, Zhi-Wen Liang, Jun Han, Jian-Ping Bi, Zhi-Yong Yang, Hong Ma

**Affiliations:** Cancer Center of Union Hospital, Tongji Medical College, Huazhong University of Science and Technology, No. 13 Hangkong Road, Wuhan, Hubei 430022 China

**Keywords:** Double-arc volumetric modulated arc therapy, Intensity-modulated radiotherapy, Three-dimensional conformal radiotherapy, Gastric cancer

## Abstract

**Background:**

The objective of this study was to compare the dose distributions of RapidArc (RA), static gantry intensity-modulated radiotherapy (IMRT), and three-dimensional conformal radiotherapy (3DCRT) as adjuvant radiotherapy modalities for the treatment of gastric cancer.

**Methods:**

Fifteen patients with gastric cancer that underwent limited lymphadenectomy of perigastric lymph nodes were included in this study. Dosimetric values for a total dose of 45 Gy (1.8 Gy/day) were calculated for the RapidArc, IMRT, and 3DCRT modalities. The following parameters were compared: D_99%_, D_1%_, V_95%,_ V_107%_, and conformity and homogeneity index values (CI and HI, respectively) for the planned target volume (PTV). Dose volume histogram (DVH) and dose distribution of the organs at risk (OAR), as the maximal dose to the spinal cord, V_30_ and V_40_ of the small bowel, and V_20_, V_30_ of liver and kidney were also assessed respectively.

**Results:**

RA, IMRT, and 3DCRT all achieved desirable PTV coverage. However, RA and IMRT significantly decreased D_1%_ and V_107%_, and provided better CI and HI values compared with 3DCRT (*P* <0.05). Moreover, RA also achieved a significantly lower maximum dose for the spinal cord, liver V_30_, and kidney V_20_ compared to IMRT and 3DCRT; while the mean dose for these three organ types did not differ for the RA, IMRT, and 3DCRT plans.

**Conclusions:**

Both RA and IMRT achieved favorable PTV coverage compared to 3DCRT. In addition, RA achieved better dosimetry than IMRT and 3DCRT, and provided better protection for the spinal cord, liver, and kidneys.

## Background

The INT0116 study revealed the survival benefits of postoperative radiotherapy for gastric cancer patients [1, 2]. Moreover, both the 3-year and 11-year follow-up results affirmed the overall survival and disease-free survival benefits of radiotherapy [1, 2]. Despite these results, however, gastric cancer radiotherapy remains controversial. Specifically, concerns remain regarding radiation-induced toxicity. The toxicity levels reported for the INT0116 study included grade 3 (40 %), grade 4 (32 %), and gastrointestinal toxicity (33 %), and three cases involved treatment-related deaths. Consequently, treatment-related toxicity remains a limiting factor for the application of gastric cancer radiotherapy [1].

In recent years, three-dimensional conformal radiotherapy (3DCRT) and intensity modulated radiotherapy (IMRT) have been widely used for the treatment of cancer. These techniques address the drawbacks of conventional anteroposterior-posteroanterior techniques, such as under-dosage of target regions and excessive radiation to surrounding normal structures. An advantage of IMRT technology over 3DCRT for the treatment of nasopharyngeal carcinoma, prostate cancer, and lung cancer has been improved dose distribution within the target area, better dose hotspot control, and reduced radiation exposure to organs at risk (OAR), including the brain stem and spinal cord [3–5]. However, it continues to be debated whether IMRT or 3DCRT is better for gastric cancer radiotherapy [6, 7]. In our previous study, IMRT was found to provide better target uniformity and conformality than four-field 3DCRT. However, IMRT did not reduce the dose applied to the OAR (e.g., the liver and kidneys) [8]. Therefore, the availability of new technologies is of great interest.

RapidArc (RA) is a type of dynamic IMRT that involves application of a rotation beam according to Otto’s rotation theory of intensity-modulated radiation therapy. Briefly, by dynamically changing the gantry rotation speed, the shape of the multi collimator leaves, and the dose rate, RA can rapidly and efficiently achieve superior radiation dose distribution [9]. As such, RA technology has the potential to shorten treatment time and reduce the possibility of target movement during treatment, thereby increasing treatment accuracy. Currently, the literature available regarding RA mainly focuses on the treatment of breast, prostate, and lung cancer [10–12]. In contrast, only a few studies have reported clinical applications of RA for gastric cancer [13].

To date, neither 3D-CRT nor IMRT have shown a clear advantage in gastric cancer radiotherapy. This is mostly attributed to the extensive region of the OAR that is involved. It also remains to be determined whether RA technology would improve the outcome of gastric cancer radiotherapy. Therefore, the goal of this study was to compare the dose distribution of RA, static gantry IMRT, and 3DCRT for the radiotherapy treatment of gastric cancer using dosimetric analysis, and to evaluate which external radiation technology is best for the postoperative treatment of gastric cancer.

## Methods

### Patients

Between October 2010 and December 2011, 15 gastric cancer patients who underwent D1 surgery at our hospital were enrolled in this study. According to the 2010 AJCC staging manual for gastric cancer [14], there were 6 T3 stage patients and 9 T4 stage patients. In addition, the lymph nodes in 7/9 patients were negative. The primary tumors were located in the cardia (*n* = 4), the pylorus (*n* = 6), or in the gastric body (*n* = 5). For this retrospective study, all of the patients completed 3D-CRT treatment before December 2011. Based on the CT images that were collected, three different treatment plans (3DCRT, IMRT, and RapidArc) were generated in order to compare the dose distributions of each. This study was approved by the ethics committee of the hospital and informed consent was obtained from all of the patients.

### Patient positioning

Each patient achieved a supine position with their hands over their chest. This position was then fixed using a thermoplastic mask. Patients fasted 4 h before the simulation computed tomography (CT) scans, and they were administered iohexol in 200 ml water orally 10 min before positioning. Enhanced CT scans were performed with a slice thickness of 3 mm. CT images were transferred to the Aria Network (Varian system) and were reconstructed using the ECLIPSE treatment planning system (Version 11, Varian Medical System, Palo Alto, CA, USA).

### Target and OAR delineation

According to Report 62 [15] of the International Commission on Radiation Units and Measurements that refers to CT and other imaging methods, clinical target volume (CTV) included the anastomosis, tumor bed, and regional lymph nodes. The planning target volume (PTV) was defined as a uniform 5 mm expansion of the CTV. The liver, left kidney, right kidney, spinal cord, small intestine, heart, and other OAR were delineated step-by-step as previously described [16].

### Treatment planning

Three radiotherapy treatment plans were generated using the Varian Eclipse treatment planning system (Version 11, Varian Medical System, Palo Alto, CA, USA) by an experienced physicist. For each of the plans, 6 MV photon beams from a Trilogy machine (Varian Medical System) were used and dose calculations were performed using the Acrous XB algorithm. For 4-field 3DCRT, the center of the PTV was designated the center of the irradiation field. The box technique in 3DCRT was found to better protect the OAR compared with half beam techniques and the use of wedges. Therefore, in this study, the box technique was selected and the incident angles used were 0°, 90°, 180°, and 270°. The dose applied at the center of the central plane was also set as the reference. For fixed-field sliding window IMRT, the gantry angle was fixed at 0°, 35°, 90°, 180°, and 315°. For RA, the coplanar double arc included 358° of rotation therapy, with 179° as the starting angle and 330° as the end angle. A maximum dose rate of 600 monitor units (MU)/min was applied. For all three plans, the prescribed dose for the PTV was 45 Gy/25 F. This dose was established to ensure that >95 % of the PTV received 45 Gy, and 99 % of the PTV received >42.75 Gy. For the OAR, less than 30 % of the whole liver volume was allowed to receive > 30 Gy (V_30_ ≤30 %). For the contralateral kidney, the volume exposed to more than 20 Gy was also limited to <30 % (V_20_ <30 %). The allowed mean dose (D_mean_) for each kidney was <18.0 Gy, and the maximum allowed dose for the spinal cord was <45 Gy. Radiation exposure to the small intestine was also minimized during the generation of the radiotherapy treatment plans. The V_40_ and V_25_ for the heart were <30 % and <50 %, respectively [16, 17].

### Evaluation and comparison of the three treatment plans

Dose distributions to the target organs and the OAR for the fifteen patients were assessed. Dose volume histograms (DVH) were also generated and compared, with the specific dosimetry parameters evaluated as follows:

To evaluate target coverage, the dose received by 99 % and 1 % of the volume (e.g., D_99%_ and D_1%_, respectively) were defined as metrics for the minimal and maximal doses [18]. The volumes receiving at least 95 % and 107 % of the prescribed dose (V_95%_ and V_107%_, respectively), as well as target homogeneity and conformal index values (HI and CI, respectively), were also compared. HI was calculated as: HI = (D2–D98)/D50. The greater the HI value, the poorer the uniformity of the dose distribution [19]. CI was calculated as follows: V_T, ref_/V_T_ × V_T, ref_/V_ref_, where V_T,ref_ is the volume of target covered by the reference isodose line, V_T_ is the target volume (= PTV), and V_ref_ is the volume of tissue covered by the reference isodose line. The value of CI varies between 0 and 1, and a value closer to one indicates better conformity of dose to the PTV [20, 21]. The equivalent uniform dose (EUD) for each PTV was compared.

Dose distribution to vital organs, including kidneys, liver, small intestine, and spinal cord, were also assessed. The parameters that were compared included mean dose (D_mean_) and V_20_ for the kidneys (V_20_ is the percentage volume of the kidneys that received at least 20 Gy), D_mean_ and V_30_ for liver, V_30_ and V_40_ for small intestine, and maximum dose (D_max_) and D_1%_ for the spinal cord. Monitor units (MU) were also compared between three plans.

### Statistical analysis

SPSS (version 17.0, SPSS Inc., Chicago, IL, USA) was used for data analysis. Non-parametric Wilcoxon tests or two-tailed *t*-tests were performed to compare groups. A *p*-value less than 0.05 was considered statistically significant.

## Results

### PTV comparison

**Fig. 1:**
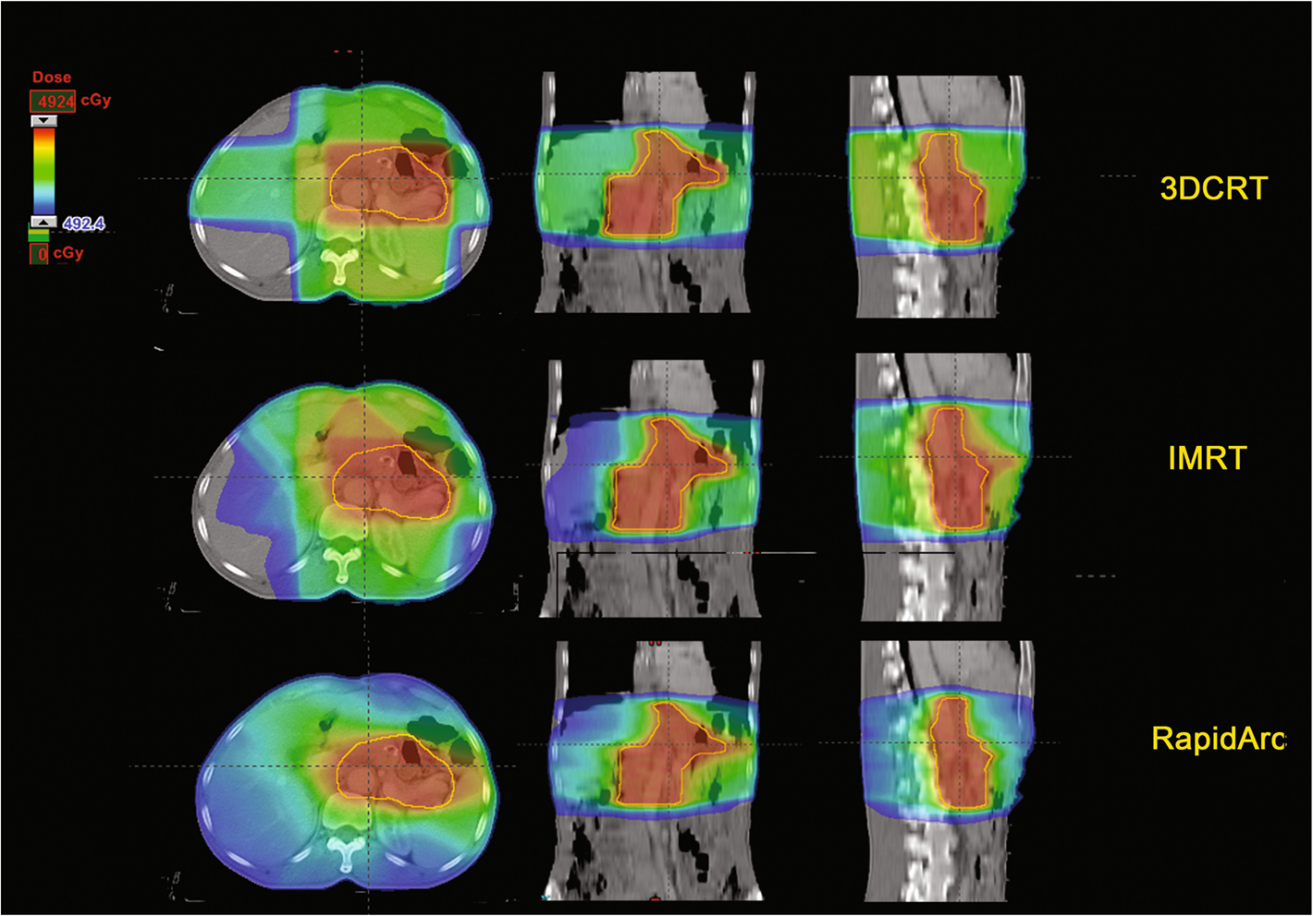
Comparison of the PTV isodose distributions achieved with 3DCRT, IMRT, and RapidArc adjuvant radiotherapy modalities

**Fig. 2:**
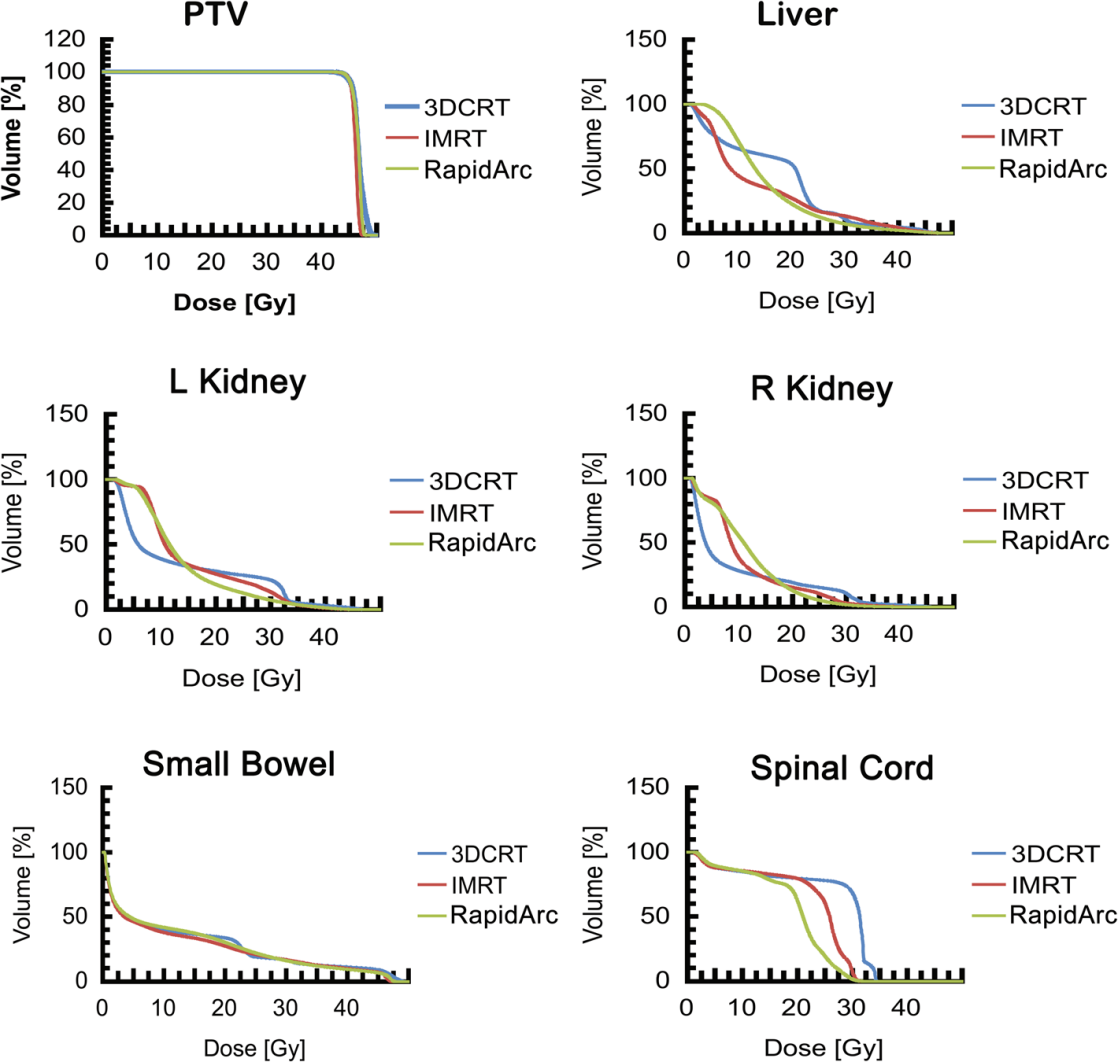
Mean dose volume histograms for PTV, CTV, OAR, and healthy tissue for global analysis according to treatment plan. 3DCRT: 3D conformal radiation therapy (blue); IMRT: intensity-modulated radiation therapy (red); RapidArc: double-arc RapidArc (green)

All three plans fulfilled the dose requirement, and there were no significant differences in the D_99%_ minimum dose and the V_95%_ target volume between them. However, the RA plan did significantly reduce the maximum target dose and the high dose volume (e.g., D_1%_ and V_107%_, respectively) compared to the 3DCRT and IMRT plans, and the difference between the 3DCRT and IMRT plans was not significant. Both IMRT and RA significantly lowered the EUD of the PTV compared to 3DCRT (*P* <0.05). Regarding target uniformity, both IMRT and RA also improved the PTV uniformity compared to 3DCRT (*P* <0.05). In addition, the CI values were 0.91 ± 0.02 for RA, 0.89 ± 0.04 for IMRT, and 0.71 ± 0.01 for 3DCRT. The former was significantly closer to a value of 1 compared with the IMRT and 3DCRT plans (*P* <0.05) (Tables 1 and 2; Figs. 1 and 2).

**Table 1:** Summary of the DVH parameters examined

Parameters		3DCRT Mean ± SD	IMRT Mean ± SD	RA Mean ± SD
PTV	D_1%_ (Gy)	49.9 ± 0.29	48.2 ± 0.17	48.4 ± 0.24
	D_99%_ (Gy)	40.5 ± 0.43	40.3 ± 0.41	40.1 ± 0.37
	V_95%_ (%)	96.6 ± 0.61	98.9 ± 0.33	98.5 ± 0.62
	V_107%_ (%)	13.6 ± 3.7	1.02 ± 0.01	1.02 ± 0.01
	EUD (Gy)	47.3 ± 0.09	46.8 ± 1.45	46.4 ± 0.06
	HI	0.1 ± 0.01	0.05 ± 0.01	0.07 ± 0.01
	CI	0.71 ± 0.02	0.89 ± 0.04	0.90 ± 0.02
Normal liver	D_mean_ (Gy)	17.6 ± 0.82	14.2 ± 0.73	15.3 ± 1.1
	V_30_ (%)	12.3 ± 1.6	12.7 ± 1.3	6.90 ± 1.4
Left kidney	D_mean_ (Gy)	13.2 ± 1.21	15.3 ± 0.63	14.1 ± 0.61
	V_20_ (%)	29.9 ± 2.5	27.7 ± 1.8	22.4 ± 3.6
Right kidney	D_mean_ (Gy)	11.9 ± 1.4	13.5 ± 0.65	12.2 ± 0.90
	V_20_ (%)	19.2 ± 1.1	16.2 ± 1.1	12.7 ± 1.3
Small bowel	D_mean_ (Gy)	13.1 ± 0.83	12.4 ± 0.39	13.1 ± 0.76
	V_30_ (%)	17.2 ± 0.61	16.5 ± 0.67	15.6 ± 0.83
	V_40_ (%)	11.0 ± 0.38	9.78 ± 0.93	9.15 ± 0.44
Spinal cord	D_1%_ (Gy)	33.0 ± 0.74	31.0 ± 0.29	27.8 ± 0.75
MU		250 ± 3.4	694 ± 3.9	399 ± 6.8

Abbreviations: 3DCRT: 3D conformal radiation therapy; IMRT: intensity-modulated radiation therapy; RA: RapidArc; PTV: planned tumor volume; D_n%_: dose received by n% of the volume; Gy: Gray (unit); Vx%: the volume receiving ≥ x% of the prescribed dose; EUD: equivalent uniform dose; HI: homogeneity index; CI: conformity index; D_mean_: the mean dose for the organ; V_n_: the volume receiving n dose of radiation (Gy); MU: monitor units; EUD: equivalent uniform dose

**Table 2:** Differences among the three methods with regard to the DVH parameters

Parameters		*P*-values			
		Overall	3DCRT vs. IMRT	3DCRT vs. RA	IMRT vs. RA
PTV	D_1%_ (Gy)	0.003	3DCRT > IMRT**	3DCRT > RA**	−
	D_99%_ (Gy)	0.803	−	−	−
	V_95%_ (%)	0.533	−	−	−
	V_107%_ (%)	0.005	3DCRT > IMRT*	3DCRT > RA*	−
	EUD (Gy)	0.012	3DCRT > IMRT*	3DCRT > RA*	_
	HI	0.03	3DCRT > IMRT*	3DCRT > RA*	−
	CI	0.001	3DCRT < IMRT*	3DCRT < RA*	−
Normal liver	D_mean_ (Gy)	0.058	3DCRT > IMRT**	−	−
	V_30_ (%)	0.006	−	3DCRT > RA**	IMRT > RA**
Left kidney	D_mean_ (Gy)	0.335	−	−	−
	V_20_ (%)	0.137	−	3DCRT > RA*	−
Right kidney	D_mean_ (Gy)	0.912	−	−	−
	V_20_ (%)	0.005	−	3DCRT > RA**	−
Small bowel	D_mean_ (Gy)	0.657	−	−	−
	V_30_ (%)	0.075	−	−	−
	V_40_ (%)	0.453	−	−	−
Spinal cord	D_1%_ (Gy)	0.011	−	3DCRT > RA*	IMRT > RA*
MU		0.001	IMRT > 3DCRT**		IMRT > RA**

Abbreviations: 3DCRT: 3D conformal radiation therapy; IMRT: intensity-modulated radiation therapy; RA: RapidArc; PTV: planned tumor volume; D_n%_: dose received by n% of the volume; Gy: Gray (unit); Vx%: the volume receiving ≥ x% of the prescribed dose; EUD: equivalent uniform dose; HI: homogeneity index; CI: conformity index; D_mean_: the mean dose for the organ; V_n_: the volume receiving n dose of radiation (Gy); MU: monitor units

*P <0.05; **P <0.01

### Evaluation of OAR

Previous research has shown that the D_mean_ and V_30_ for the liver are important predictors of radiation-induced liver damage [8]. In the present study, V_30_ for the liver was (12.32 ± 1.61) % for 3DCRT, (12.73 ± 1.33) % for IMRT, and (6.90 ± 1.41) % for RA. The latter was significantly lower than the other two methods (*P* <0.05). In addition, the D_mean_ for the liver was 17.61 ± 0.82 Gy for 3DCRT, 14.22 ± 0.23 Gy for IMRT, and 15.31 ± 1.11 Gy for RA. Both IMRT and RA reduced the average radiation dose for the liver, yet the difference was not significant (Tables 1 and 2; Fig. 2). In a previous study by Matzinger and Dawson [16, 17], the recommended tolerance doses for the kidneys were V_20_ <30 % and D_mean_ <18 Gy. In the present study, the V_20_ for the left or right kidney with the RA plan was lower than that for the IMRT and 3DCRT plans. Specifically, RA treatment decreased the V_20_ of the left kidney by 25.17 %, and the right kidney by 33.94 %. In addition, the average dose for both kidneys was higher for both the IMRT and RA plans compared to 3DCRT, although the difference was not significant (*P* >0.05) (Tables 1 and 2; Fig. 2).

The V_30_ and V_40_, as well as the D_mean_, for the small intestine were also assessed. Compared with 3DCRT, IMRT and RA only moderately reduced V_30_ and V_40_, and the differences were not significant. In contrast, RA slightly increased the D_mean_ of the small intestine, although this difference was also not significant (*P* >0.05) (Tables 1 and 2; Fig. 2).

For the spinal cord, all three plans fulfilled the dose requirements. The maximum radiation doses for D_1%_ were 32.98 ± 0.74 Gy for 3DCRT, 31.01 ± 0.29 Gy for IMRT, and 27.80 ± 0.75 Gy for RA. Compared with 3DCRT, RA significantly lowered the spinal cord D_max_ value by 15.71 % (Tables 1 and 2; Fig. 2).

### Comparison of MU and delivery parameters

The MU for IMRT and RA were 694.25 ± 3.91 and 399.00 ± 6.81, respectively. Thus, RA significantly reduced the radiation dose received by 42.5 % compared with IMRT. However, RA required a greater number of MU than 3DCRT. In addition, a physical wedge was not used for the 3DCRT treatments since this could increase the number of MU, and it could also increase the potential for leakage radiation (Tables 1 and 2). The dose rate for each technique was 400 MU/min for 3DCRT, 600 MU/min for IMRT, and nearly 600 MU/min for ARC. Thus, the relative treatment times for each technique were: 3.2 ± 0.3 min for RA, 6.6 ± 1.2 min for IM, and 4.2 ± 0.5 min for CRT.

## Discussion

Currently, postoperative chemoradiation is one of the main treatments for cases of gastric cancer with poor prognosis. However, due to the proximity of this region to many vital organs, it remains a challenge to effectively cover the target area and protect neighboring vital organs. For adjuvant radiotherapy modalities, it has been difficult to achieve an ideal dose distribution with traditional 3DCRT, while IMRT is able to simultaneously optimize target dose and decrease exposure of OAR. IMRT has also been shown to effectively improve local tumor control, to reduce the extent of radiation damage to normal tissues, and to improve patient quality of life [22]. However, in our previous study of 3DCRT and IMRT for the treatment of gastric cancer, radiation dosimetry data indicated that IMRT did not show a significant advantage over 3DCRT, with 3DCRT being superior to IMRT for V_20_ of the left and right kidneys [8]. Therefore, new radiotherapy techniques for the treatment of gastric cancer are still needed.

RA technology has the potential to shorten treatment time and reduce the possibility of target movement during treatment, which would serve to increase treatment accuracy [18]. RA has previously been applied to the treatment of many types of tumors [23, 24]. For example, in work by Verbakel et al. [25], twelve patients with advanced head and neck cancer received IMRT versus RA radiation therapy. Treatment with RA was found to improve target dose uniformity and to reduce exposure of neighboring OAR. Furthermore, double arc RA provided additional dosimetric advantages compared to single arc RA and IMRT. These advantages were confirmed with the treatment of lung cancer and prostate cancer with double arc RA [11, 12]. However, for gastric cancer radiation therapy, the shape of the radiation target is irregular and the surrounding organs, including the liver and kidneys, have a low tolerance for radiation. Thus, it remains to be determined whether a rotary volumetric IMRT technique will be advantageous for gastric cancer radiotherapy.

The cohort studied included 15 postoperative gastric cancer patients. Based on the location of their lesions and CT imaging, 3DCRT (4-field), IMRT (5-field), or RA treatment plans were applied. The prescription dose included 45 Gy/25 F applied to the PTV, with >95 % of the PTV receiving 45 Gy and 99 % of the PTV receiving 42.75 Gy. All three plans met the dose requirements and there were no significant differences between them. Furthermore, IMRT and RA reduced the target maximum dose and the high dose range (D_1%_, V_107%_) compared to 3DCRT. IMRT and RA were also superior to 3DCRT for target volume uniformity. The CI value for RA was significantly closer to one than the CI values for IMRT and 3DCRT, suggesting an improved conformality was achieved. For targets with larger and more complex shapes, RA was found to provide better dose distribution, better PTV target conformality, and better target dose distribution, and these results are consistent with previous studies [13]. Thus, RA has the potential to reduce treatment-related side effects.

In the early studies of organ tolerance to ionizing radiation, radiosensitivity of the liver may have been underestimated. Tolerance doses were limited according to the risk of RT-induced liver disease, and the mean dose and V30 for the liver were considered important dosimetric parameters associated with increased toxicity risk [26]. Meanwhile, more recent studies have shown that normal liver cells are sensitive to radiation, especially when the liver is infected with hepatitis B virus [26]. Accordingly, Dawson et al. [27] have suggested that the tolerance dose for the liver should be less than 30 % for V_30_, and the D_mean_ should be less than 30 Gy. For cases involving hepatitis B infection, the D_mean_ should be less than 23 Gy. Furthermore, according to the Quantitative Analyses of Normal Tissue Effects in the Clinic (QUANTEC) effort, the mean liver dose should be less than 28 Gy in 2-Gy fractions for primary liver cancer, and should be less than 32 Gy in 2-Gy fractions for liver metastases [26]. In the present study, liver V_30_ was (12.32 ± 1.61) % for 3DCRT, (12.73 ± 1.33) % for IMRT, and (6.90 ± 1.41) % for RA, with the latter being significantly lower than the two former values (*P* <0.05). Liver D_mean_ was 17.61 ± 0.82 Gy for 3DCRT, 14.22 ± 0.23 Gy for IMRT, and 15.31 ± 1.11 Gy for RA, and these did not significantly differ. Compared with 3DCRT and IMRT, RA significantly reduced liver V_30_, yet did not affect the average liver dose. Furthermore, despite the significant reduction in liver V_30_, an analysis of volume from the DVH showed that V_10_ increased. These results are consistent with those reported for a liver cancer radiation treatment study performed by Kuo et al. [28].

The kidney is another important organ that is threatened by gastric cancer radiotherapy. Kidney tissue is radiosensitive, and the recommended radiation tolerance doses are 23 Gy for the whole kidney, 30 Gy for 2/3 of the kidney, and 50 Gy for 1/3 of the kidney. A study by Jansen et al. further suggested that the average renal dose was less important than V_20_. Therefore, it is recommended that <70 % of the kidney volume should receive 20 Gy (V_20_ <70 %), while the V_20_ for the contralateral kidney should be <30 % [29]. In total, kidney tissues exposed to more than 20 Gy should not exceed 50 % of the whole kidney, otherwise, radiation-induced damage to the kidney may occur, such as a decrease in the glomerular filtration rate and/or renal failure. Thus, an ongoing goal is to reduce the radiation dose to kidneys during postoperative radiotherapy for gastric cancer. Minn et al. [30] studied the dosimetry, efficacy, and toxicity of radiotherapy planning with 3DCRT and IMRT for 57 cases of gastric cancer, and IMRT was found to reduce kidney V_20_. In our previous study, no obvious difference in the V_20_ of kidney between IMRT and 3D-CRT was observed, although IMRT exhibited favorable tumor coverage and superiority in protecting the spinal cord and liver. However, this superiority was not observed in the kidney compared with 3D-CRT. Thus, IMRT does not appear to represent a superior treatment for gastric cancer [8]. Similarly, in our subsequent single arc RA study, kidney radiation dose was not significantly reduced, yet double arc RA significantly decreased kidney V_20_ compared to IMRT and 3DCRT for both kidneys. Meanwhile, there was no obvious difference in the D_mean_ for both kidneys among the 3D-CRT, IMRT, and RA treatments. Taken together, these results suggest that RA can provide a protective effect for kidneys compared to IMRT.

Gastrointestinal toxicity is the main limiting factor for the application of radiation therapy to gastric cancer. Correspondingly, the key to reducing toxicity due to radiotherapy is to control the exposure of the gastrointestinal tract to radiation. In many studies, IMRT and RA have been shown to reduce the radiation dose to the gastrointestinal tract during abdominal radiation therapy. For example, Minn et al. [30] demonstrated that IMRT reduced intestinal V_45_ compared to 3DCRT. In another study of 14 cases of abdominal metastases treated with radiation therapy, Mario et al. [30] reported that RA and IMRT reduced the average dose and maximum dose to the stomach and small intestine compared to 3DCRT. However, the difference was not significant. In the present study, the average dose (D_mean_) for the small intestine, as well as V_30_ and V_40,_ were examined. D_mean_ for the small intestine did not significantly differ among the three planning methods, yet a DVH chart analysis showed that IMRT and RA increased V_10_ and reduced V_30_ and V_40_ compared to 3DCRT. Thus, the volume of the low dose region increased concomitant with a decrease in the volume of the high dose region. These results are consistent with the observation that the average dose did not show a significant difference.

The spinal cord is a long, thin, tubular bundle of nervous tissue and it is susceptible to injury from local high doses of radiation. Kirkpatrick et al. [31] reported that the incidence rate for radiation myelitis is 0.2, 6, and 50 % the total dose for 50 Gy, 60 Gy, and_69 Gy, respectively, when administered at the conventional fraction of 2-Gy per day. In addition, according to the Radiation Oncology Group of the European Organisation for Research and Treatment of Cancer, the maximal radiation dose that should be applied to the spinal cord is 45 Gy, and it should not exceed 40 Gy if oxaliplatin chemotherapy is administered as well [16]. Therefore, the maximum dose for the spinal cord is generally set at no more than 45 Gy. In the present study, the doses applied to the spinal cord with each of the three techniques were all within the tolerated dose. Moreover, compared to 3DCRT, the D_max_ for the spinal cord with RA was significantly reduced by up to 15.71 %.

RA is an adjuvant radiotherapy modality that has recently been developed and has been used to deliver high doses of radiation to a variety of tumors. However, its role in the treatment of gastric cancer remains controversial due to the irregular target volumes involved and the low radiation tolerance of surrounding critical organs. In our previous study, RA provided superior dose homogeneity compared with 3DCRT and IMRT, but not better protection of the OAR. Moreover, while the single arc technique was unsuccessful, the double arc technique was able to achieve the same dose distribution as IMRT, while significantly sparing the OAR and proximal healthy tissue. This improved protection of liver and kidney tissues compared with IMRT suggests a higher dose could be applied to a target volume using double arc RA. However, it is important to consider the limitations of our study as well. First, a respiratory gating technique was not used, and its influence on the dose distribution was not investigated. In addition, the present study had a small sample size and did not evaluate clinical efficacy and toxicity. Therefore, further studies are needed to confirm the technical feasibility of applying double arc RA to the treatment of gastric cancer, and these should include a larger sample size and evaluations of clinical efficacy and toxicity.

## Conclusions

In summary, double arc RA reduced the maximum dose applied to the target area, it improved the conformality and uniformity of radiation, and it provided sufficient PTV coverage. Moreover, compared to 3DCRT and IMRT, double arc RA significantly reduced liver V_30_, kidney V_20_, and the spinal cord maximum dose. Furthermore, RA was found to provide the best protection from high doses of radiation. Therefore, in this comparative study, a theoretical foundation for the clinical application of radiation for gastric cancer is demonstrated. Despite the small sample size, the results are very promising and further studies are needed to confirm these results.

## Abbreviations

IMRT: Intensity-modulated radiotherapy
3DCRT: Three-dimensional conformal radiotherapy
PTV: Planned target volume
DVH: Dose volume histogram
OAR: Organs at risk
RA: RapidArc
CT: Computed tomography
CTV: Clinical target volume
